# Early Onset of Postoperative Gastrointestinal Dysfunction Is Associated With Unfavorable Outcome in Cardiac Surgery: A Prospective Observational Study

**DOI:** 10.1177/0885066620946006

**Published:** 2020-08-10

**Authors:** Jenny Seilitz, Måns Edström, Martin Sköldberg, Kristian Westerling-Andersson, Alhamsa Kasim, Anja Renberg, Kjell Jansson, Örjan Friberg, Birger Axelsson, Kristofer F. Nilsson

**Affiliations:** 1 Department of Cardiothoracic and Vascular Surgery, Faculty of Medicine and Health, Örebro University, Örebro, Sweden; 2 Department of Anaesthesiology and Intensive Care, Faculty of Medicine and Health, Örebro University, Örebro, Sweden; 3 Department of Surgery, Faculty of Medicine and Health, Örebro University, Örebro, Sweden

**Keywords:** cardiac surgery, mortality, outcome, complications, gastrointestinal tract

## Abstract

**Objective:**

The distribution of postoperative gastrointestinal (GI) dysfunction and its association with outcome were investigated in cardiac surgery patients. Gastrointestinal function was evaluated using the Acute Gastrointestinal Injury (AGI) grade proposed by the European Society of Intensive Care Medicine.

**Design:**

Prospective observational study at a single center.

**Setting:**

University hospital.

**Patients:**

Consecutive patients presenting for elective cardiac surgery with extracorporeal circulation (ECC).

**Interventions:**

None.

**Results:**

Daily assessment using the AGI grade was performed on the first 3 postoperative days in addition to standard care. For analysis, 3 groups were formed based on the maximum AGI grade: AGI 0, AGI 1, and AGI ≥2. Five hundred and one patients completed the study; 32.7%, 65.1%, and 2.2% of the patients scored a maximum AGI 0, AGI 1, and AGI ≥2, respectively. Patients with AGI grade ≥2 had more frequently undergone thoracic aortic surgery and had longer surgery duration and time on ECC. Patients with AGI grade ≥2 had statistically significant higher frequency of GI complications within 30 days (63.6% vs 1.2% and 5.5% in patients with AGI 0 and AGI 1) and higher 30-day mortality (9.1% vs 0.0% and 1.8% in patients with AGI 0 and AGI 1).

**Conclusions:**

Early GI dysfunction following cardiac surgery was associated with an unfavorable outcome. Increased attention to GI dysfunction in cardiac surgery patients is warranted and the AGI grade could be a helpful adjunct to a structured approach.

## Introduction

In 2012, the European Society of Intensive Care Medicine proposed the Acute Gastrointestinal Injury (AGI) grade to help define, grade, and guide the treatment of gastrointestinal (GI) problems in the intensive care unit (ICU).^1^ Four different AGI grades were defined: AGI 1: a self-limiting condition with future risk of developing GI dysfunction, AGI 2: dysfunction of the GI tract where interventions are required to restore GI function, AGI 3: GI failure despite interventions, and AGI 4: life-threatening GI failure affecting other organs. A higher AGI grade in critically ill patients has been associated with increased mortality.^2,3^

Gastrointestinal complications after cardiac surgery occur in approximately 1.2% of cases and are associated with high mortality.^4–7^ The most common GI complications are postoperative ileus and GI bleeding, while mesenteric ischemia and intestinal perforation are associated with the highest mortality rates.^4^ Mild GI symptoms, such as postoperative nausea and vomiting (PONV), are common following cardiac surgery.^8,9^ A strategy to avoid GI complications and related mortality in cardiac surgery patients could be early recognition and management of GI dysfunction since this may precede complications.

The aims of this study were to investigate the distribution of AGI grade in postoperative cardiac surgery patients and to analyze the association between AGI grade and clinical outcome, measured as diagnosis of any GI complication and mortality within 30 days postoperatively. The hypothesis was that early onset of GI dysfunction in cardiac surgery patients is associated with postoperative unfavorable outcome, especially GI complications.

## Materials and Methods

Approval was obtained from the Regional Ethical Review Board, Uppsala, Sweden (registration number 2015/365). Adult patients scheduled for elective cardiac surgery with extracorporeal circulation (ECC) at the department of cardiothoracic and vascular surgery, Örebro University Hospital, Sweden, between November 16, 2015, and October 25, 2017, were evaluated for participation. Written informed consent was obtained at inclusion. Exclusion criteria were previous major abdominal surgery, existing ostomy, liver failure, GI malignancy, current or recent GI bleeding, inflammatory bowel disease, GI arteriopathy, and/or debilitating irritable bowel syndrome. Patients included in an interventional study and those with inadequate communication skills were also excluded. This was an explorative study using a recently described scoring system in a new patient population. Therefore, we based study size estimation on previously demonstrated incidence of GI complications. The study aimed to include around 500 patients to acquire approximately 10 patients with a severe GI complication.

### Protocol

Information was obtained from the patients themselves, medical personnel, medical records, and/or a local quality register in which mortality data are synchronized with the Swedish Population Register. During the first 3 postoperative days, the patients were assessed daily using the AGI grade and GI symptoms were noted. Patients with no GI symptoms were assigned AGI grade 0, otherwise no modifications were made. Apart from the use of the AGI grade, the patients received standard care.

The occurrence of GI complications was determined by review of medical records and the diagnosis set by the treating physician. However, paralytic ileus was defined as clearly symptomatic patients remaining in the ICU with failure of bowel function for 3 consecutive days^1,10^ and GI bleeding as any bleeding into the GI tract lumen,^1^ with a decreasing hematocrit in combination with a positive test for fecal hemoglobin as the least severe presentation. In parallel with AGI scoring, other organ dysfunctions were followed. Respiratory dysfunction was defined as the need of mechanical respiratory support, continuous or intermittent, during the first to third postoperative days. This parameter was further stratified into an arterial oxygen partial pressure/fraction of inspired oxygen (PaO_2_/FiO_2_) ratio above or below 13.3 kPa. Circulatory status was evaluated through the need of vasoactive and/or inotropic drugs, continuous or intermittent, during the first to third postoperative days. Patients receiving at least 1 drug were defined as having circulatory dysfunction and subdivided into needing 1 or more than 1 drug. Neurological dysfunction was defined as the onset of neurological symptoms during the first 3 postoperative days, still present at hospital discharge. Renal dysfunction was stratified by a modified acute kidney injury (AKI) score^11^ based on plasma creatinine values and dialysis requirement during the first 3 days (AKI 1 = creatinine × 1.5-2 or ≥26.4 µM; AKI 2 = creatinine × >2-3; AKI 3 = creatinine × >3 or ≥354 μM with an acute increase of at least ≥44 μM or new dialysis).

The patients were divided into 3 groups for analysis: a maximum AGI grade of 0, 1, or ≥2 during the first 3 postoperative days (referred to as AGI_0_, AGI_1_, and AGI_≥2_, respectively). The AGI_≥2_ group was constructed post hoc due to the low number of patients receiving AGI 2, AGI 3, or AGI 4. Primary outcome variables were clinical diagnosis of any GI complication and all-cause mortality within 30 days. Secondary outcome variables were mortality within 120 and 365 days, as well as time spent on mechanical respiratory support, and length of ICU and hospital stay.

### Anesthesia and Postoperative Care: General Routines

Premedication was oral administration of 10 mg oxycodone, with the addition of 1 mg flunitrazepam as needed. General anesthesia was induced with fentanyl and propofol. After intubation, anesthesia was maintained by 0.8 to 1.3 MAC sevoflurane and repeated bolus doses of fentanyl. Depth of anesthesia was monitored using bispectral index (Covidien), with a target value of 40 to 60 and the absence of burst suppression. Infusion of propofol was used for sedation from the end of surgery until extubation. In cases of hypotension, norepinephrine was used as the first-line treatment and milrinone was the drug of choice when inotropic support was deemed necessary. Before and after ECC, the target for mean arterial pressure was 60 to 70 mm Hg. One dose of 3 g benzylpenicillin and 4 to 5 repeated doses of 2 g cloxacillin were used as perioperative antibiotic prophylaxis. The typical postoperative pain management regime consisted of oral oxycodone 10 mg twice daily and paracetamol 1 g 4 times a day, with the addition of intravenous ketobemidone as needed. Postoperatively, feeding was restarted as soon as clinically indicated, usually on the morning of the first postoperative day. For patients remaining in the ICU, the departmental routine regarding nutrition aimed to adhere to the European Society for Clinical Nutrition and Metabolism guideline.^12^ Prophylaxis against PONV was generally not used but, when administered, consisted mainly of ondansetron and/or betamethasone prior to extubation. Proton-pump inhibitors were continued if previously prescribed, used for stress ulcer prevention in selected cases at the clinician’s discretion, and given to patients in the ICU not on full enteral nutrition. First-line laxatives were an osmotic laxative (macrogol) and sodium picosulfate, while metoclopramide was used for gastroparesis.

### Extracorporeal Circulation: General Routines

Standard cannulas were used (Medtronic). The extracorporeal circuit consisted of a hollow fiber oxygenator with integral heat exchanger (Inspire 8M; LivNova), a hard-shell venous reservoir (Inspire 8 Dual; LivNova), a S5 Stöckert roller pump set (Stöckert Instrumente GmbH), and a customized, uncoated, tubing set (LivNova). The oxygenator and tubing set were primed with 1250-mL Ringer’s acetate, 250-mL mannitol (150 mg/mL), and 10 000 IU heparin. Cold blood cardioplegia was used and the solution contained 100 mM potassium, 5 mM procaine, and 80 mM magnesium. Nonpulsatile flow rate was set at 2.4 L/min/m^2^, aiming at a mixed venous oxygen saturation >70%. While on ECC, mean arterial pressure was kept at 55 to 70 mm Hg and hematocrit >25%. The target body temperature was 34 °C on ECC and 25 °C during systemic circulatory arrest with antegrade selective cerebral perfusion (ASCP). When ASCP was used, the flow rate was set at approximately 10 mL/min/kg and the temperature of the perfusate was 20 °C. Anticoagulation was achieved with heparin to maintain an activated clotting time >480 seconds. Heparin was neutralized with protamine sulfate after weaning from ECC. Tranexamic acid was given, 2 g before the initial heparin dose and 2 g after the administration of protamine sulphate.

### Statistics

The normality of data was checked by Shapiro-Wilk test. Continuous parameters were compared using Kruskal-Wallis *H* test or 1-way analysis of variance where appropriate. Dunn and Tukey Honest Significant Difference post hoc tests were used. Categorical data were analyzed using χ^2^ or Fisher exact test depending on the expected number of patients in each cell (threshold n ≤ 5). Dependent variables with more than 2 categories were analyzed separately in a one-vs-all fashion where each category was separately compared to the others, resulting in a 2 × 3 table with one dichotomous dependent variable versus AGI group. The *P* values of these separate, post hoc analyses were corrected using false discovery rate according to Benjamini and Hochberg.^13^ To aid interpretation of deviations from expected proportions, standardized residuals were calculated. Frequencies with a standardized residual of more than ±1.96 were treated as deviating from the expected count. A multivariate Cox proportional hazards regression model was used to further investigate the impact of maximum AGI grade on survival. Proportionality of hazards was assessed prior to analysis. Only preoperative characteristics were included as covariates. Throughout all statistical analyses, no data were missing. Values of *P* less than .05 were considered statistically significant. Statistical analysis was performed using IBM SPSS Statistics version 25.0 for Windows (SPSS Inc) and R version 3.5.1.^14^

## Results

**Figure 1.**
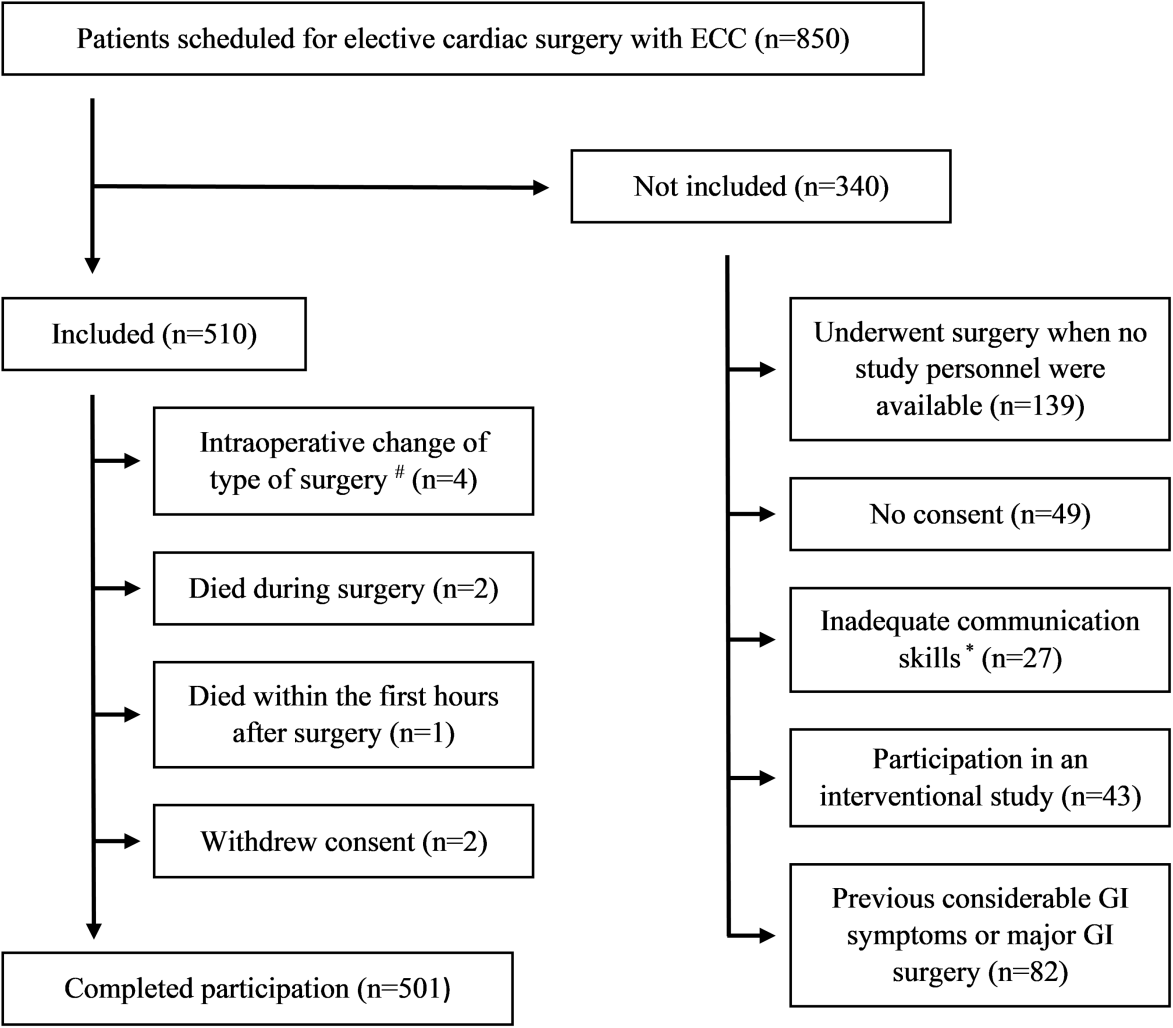
Flowchart comprising eligible study patients. ^#^ Change to off pump coronary surgery (n = 3) or transcatheter aortic valve implantation (n = 1) due to aortic calcification.^*^ Need of interpreter (n = 24), neurological or psychiatric disorder (n = 3). ECC indicates extracorporeal circulation; GI, gastrointestinal.

Of 850 patients eligible for inclusion during the study period, 510 were included and 501 completed the study (Figure 1). Three included patients died before the first AGI scoring and were excluded since AGI group allocation was not possible in these patients. No patients died within the first 3 postoperative days, that is, during the scoring period. Baseline characteristics are presented in Table 1.

**Table 1.** Baseline Characteristics and Intraoperative Variables.

Variables	Overall, n = 501	AGI 0, n = 164	AGI 1, n = 326	AGI ≥ 2, n = 11	*P*
Sex, n (%)					<.001
Female	123 (24.6)	20 (12.2%)	100 (36.7%)	3 (27.3%)	
Male	378 (75.4)	144 (87.8%)	226 (69.3%)	8 (72.3%)	
Age (years), median (IQR)	69 (62-74)	68 (61-74)	69 (62-74)	69 (51-77)	.606
BMI (kg/m^2^), median (IQR)	27.1 (24.5-30.4)	27.6 (24.9-31.0)	26.9 (24.3-30.0)	27.7 (25.3-30.2)	.415
Smoking, n (%)					.345
Never smoked	265 (52.9)	79 (48.2)	182 (55.8)	4 (36.4)	
Quit ≥1 month ago	202 (40.3)	73 (44.5)	123 (37.8)	6 (54.5)	
Active smoker	34 (6.8)	12 (7.3)	21 (6.4)	1 (9.1)	
Diabetes, n (%)					
No	385 (76.8)	114 (69.5)	262 (80.3)	9 (81.8)	.022
Oral treatment	47 (9.4)	23 (13.9)	23 (7.1)	1 (9.1)	
Insulin treatment	34 (6.8)	5 (3.0)	28 (8.6)	1 (9.1)	
Insulin and oral treatment	29 (5.8)	19 (11.8)	10 (3.1)	–	
Dietary treatment	6 (1.2)	3 (1.8)	3 (0.9)	–	
Hypertension, n (%)	327 (65.3)	112 (68.3)	206 (63.2)	9 (81.8)	.271
Heart failure, n (%)^a^	61 (12.2)	21 (12.8)	39 (12.0)	1 (9.1)	.917
Extracardiac arteriopathy, n (%)^b^	64 (12.8)	18 (11.0)	41 (12.6)	5 (45.5)	.013
EuroSCORE, median (IQR)	4 (3-6)	4 (2-6)	4 (3-6)	5 (3-8)	.254
NYHA, median (IQR)	3 (2-3)	3 (2-3)	3 (2-3)	3 (2-3)	.456
Type of surgery, n (%)					
CABG	235 (46.9)	85 (51.8)	146 (44.8)	4 (36.4)	.394
Aortic valve replacement	134 (26.7)	46 (28.0)	88 (27.0)	–	.236
CABG + aortic valve replacement	49 (9.8)	16 (9.8)	33 (10.1)	–	.978
Thoracic aortic surgery	36 (7.2)	6 (3.7)	23 (7.1)	7 (63.6)	<.001
Mitral valve surgery	33 (6.6)	6 (3.7)	27 (8.3)	–	.236
Other^c^	14 (2.8)	5 (3.0)	9 (2.7)	–	1.000
Duration of surgery (minutes), median (IQR)	231 (195-276)	236 (189-275)	226 (194-275)	295 (233-492)	.027
ECC time (minutes), median (IQR)	117 (95-142)	114 (92-139)	117 (95-145)	158 (126-258)	.022
Aortic cross-clamp time (minutes), median (IQR)	77 (63-102)	76 (62-98)	78 (64-102)	107 (63-138)	.191

Abbreviations: AGI, Acute Gastrointestinal Injury; BMI, body mass index; CABG, coronary artery bypass grafting; ECC, extracorporeal circulation; EuroSCORE, European System for Cardiac Operative Risk Evaluation; IQR, interquartile range; NYHA, New York Heart Association Functional Classification.

^a^ Diagnosed and treated medically, assessed by the attending anesthesiologist.

^b^ Claudication, carotid artery stenosis or previous or planned surgery on abdominal aorta, and carotid arteries or distal arteries or cerebral vascular disease.

^c^ Six CABG combined with mitral valvuloplasty, 3 cardiac tumors, 1 aortic valve replacement combined with mitral valve replacement, 1 tricuspid valvuloplasty combined with mitral valve replacement, 1 CABG combined with myectomy, 1 myectomy, and 1 cryoablation.

### Acute Gastrointestinal Injury Grade and GI Symptoms

**Figure 2.**
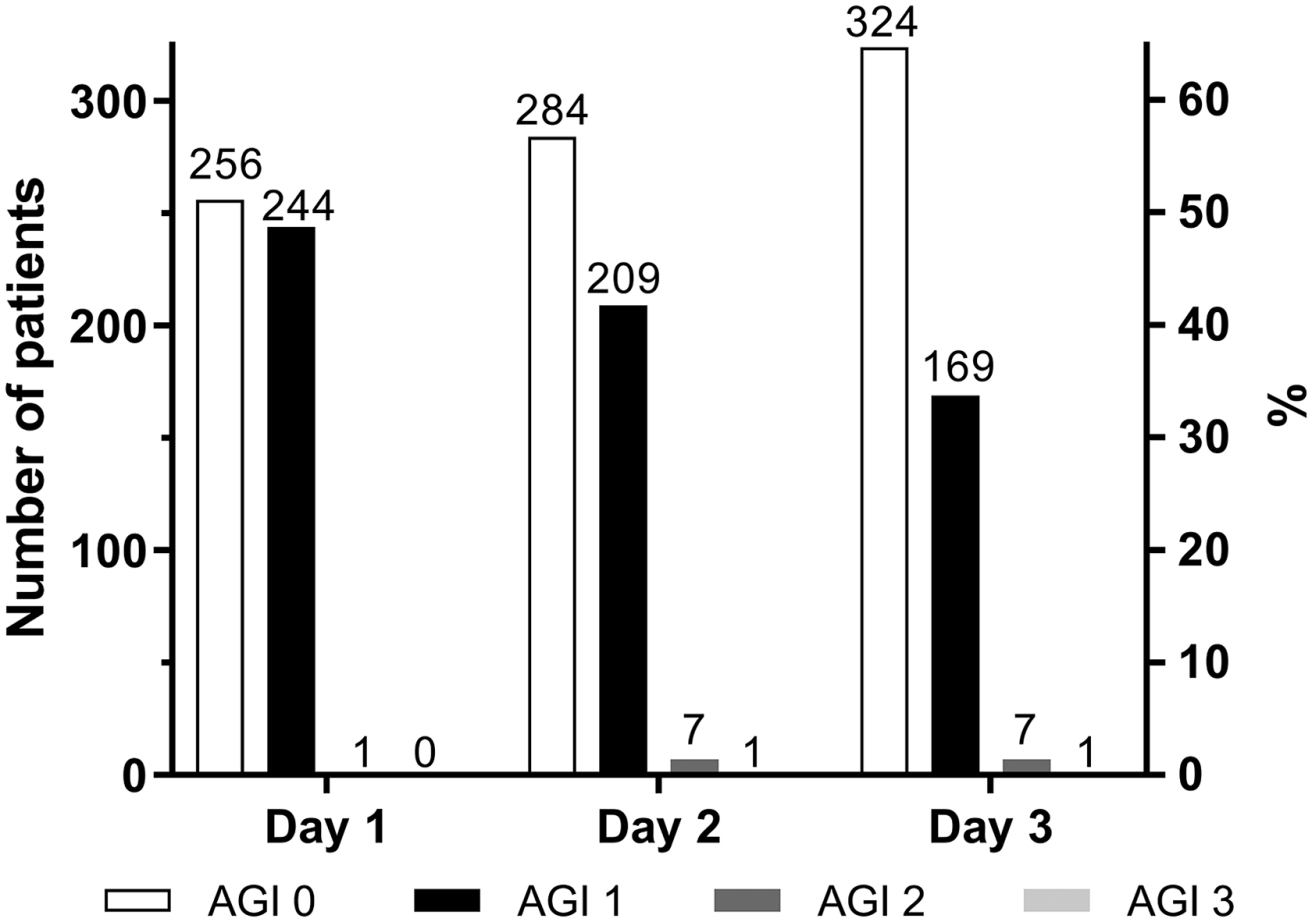
Acute Gastrointestinal Injury (AGI) grade during the 3 first postoperative days after elective cardiac surgery with extracorporeal circulation, n = 501. No patient had AGI grade 4.

The frequency of AGI grades is shown in Figure 2. Of the 501 patients who completed the study, 164 did not develop any GI symptoms, that is, had an AGI grade of 0 (32.7%), during the first 3 postoperative days. Symptoms experienced by patients with a maximum AGI grade of 1 (n = 326, 65.1%) were almost exclusively nausea and/or vomiting. Patients with a maximum AGI grade of 2 (n = 9, 1.8%) had symptoms of diarrhea, abdominal pain, aspirated gastric residual volume >200 mL, the presence of blood in the gastric aspirate, and/or food intolerance. In addition, the patients with a maximum AGI grade of 3 (n = 2, 0.4%) had increased intra-abdominal pressure (17-20 mm Hg) and abdominal circumference and were unresponsive to treatment of GI symptoms. No patient had AGI grade 4 during the first 3 postoperative days.

### Preoperative Characteristics

There was a difference in the sex distribution of patients, with fewer women in the AGI_0_ group and a higher proportion of women in the AGI_1_ group (*P* < .001; Table 1). The proportion of diabetic patients were higher in the AGI_0_ group, while the proportion was lower in the AGI_1_ group (*P* = .022). In the AGI_≥2_ group, there was a higher occurrence of extracardiac arteriopathy (*P* = .013; Table 1). There were no other statistically significant differences between the 3 AGI groups, including risk assessed using European System for Cardiac Operative Risk Evaluation (EuroSCORE; Table 1).

### Intraoperative Variables

The most frequent surgeries were coronary artery bypass grafting and aortic valve replacement (Table 1). The AGI_≥2_ group comprised more cases of thoracic aortic surgery (*P* < .001) and had longer duration of surgery and longer time on ECC (*P* = .027 and *P* = .022, respectively). In both the AGI_0_ and AGI_1_ groups, systemic circulatory arrest with ASCP was used in 1.2% of the patients versus 45.5% of those in the AGI_≥2_ group (*P* < .001). The overall median duration of ASCP was 50 minutes (range 30-113), with no statistically significant difference between the groups.

### Postoperative Organ Dysfunction

A higher proportion of patients with AGI_≥2_ was in need of mechanical respiratory support, regardless of arterial oxygen partial pressure/fraction of inspired oxygen (PaO_2_/FiO_2_) ratio (*P* = .008 and *P* = .002 for PaO_2_/FiO_2_ ≥ 13.3 kPa and PaO_2_/FiO_2_ < 13.3 kPa, respectively; Table 2). Furthermore, when assessing postoperative neurological dysfunction, with onset within the first 3 days postoperatively and still present at discharge, patients in the AGI_≥2_ group had higher occurrence (*P* = .009; Table 2). Patients with AGI ≥ 2 also received inotropic and/or vasoactive drugs after surgery to a higher degree (at least 2 drugs postoperatively, *P* = .006; Table 2) and had a higher AKI score (AKI 1, *P* = .041, AKI 2, *P* = .008, and AKI 3, *P* = .010; Table 2). In contrast, patients in the AGI_1_ group needed mechanical respiratory support postoperatively to a lower degree (*P* < .001), and patients in the AGI_0_ group displayed a lower frequency of receiving inotropic and/or vasoactive drugs (*P* = .006). No deviations from expected counts were found regarding renal dysfunction or central nervous system impairment in the AGI_0_ and AGI_1_ groups.

**Table 2.** Postoperative Organ Dysfunction and Outcome.

Variables	Overall, n = 501	AGI 0, n = 164	AGI 1, n = 326	AGI ≥ 2, n = 11	*P*
Respiratory status^a^					
PaO_2_/FiO_2_ ≥ 13.3 kPa, n (%)	25 (5.0)	10 (6.1)	12 (3.7)	3 (27.2)	.008
PaO_2_/FiO_2_ < 13.3 kPa, n (%)	3 (0.6)	–	1 (0.3)	2 (18.2)	.002
Circulatory status^b^					
One drug, n (%)	136 (27.1)	37 (22.6)	95 (29.1)	4 (36.4)	.214
Two or more drugs, n (%)	25 (5.0)	6 (3.7)	15 (4.6)	4 (36.4)	.006
Neurologic impairment,^c^ n (%)	11 (2.2)	1 (0.6)	8 (2.5)	2 (18.1)	.009
Acute kidney injury score^d^					
Creatinine × 1.5-2 *or* ≥26.4 µM, n (%)	37 (7.4)	9 (5.5)	25 (7.7)	3 (27.2)	.041
Creatinine × >2-3, n (%)	6 (1.2)	1 (0.6)	3 (0.9)	2 (18.2)	.008
Creatinine × > 3 *or* ≥354 μM with an acute increase of at least ≥44 μM *or* new dialysis, n (%)	9 (1.8)	1 (0.6)	6 (1.8)	2 (18.2)	.010
Time on mechanical respiratory support (minutes), median (IQR)	195 (150-285)	180 (150-295)	197 (150-270)^e^	4898 (200-27462)^f^	.006
ICU length of stay (hours), median (IQR)	21.0 (19.3-22.1)	21.2 (19.7-22.5)	20.8 (19.0-21.8)^e^	271.1 (18.8-603.8)^f^	<.005
Hospital length of stay (days), median (IQR)	7 (6-10)	7 (6-9)	8 (6-10)^g^	31 (6-88)^h^	<.005
Patients with GI complications within 30 days, % (95% CI)	5.4% (3.7-7.7); n = 27	1.2% (0.3-4.3); n = 2	5.5% (3.5-8.6); n = 18	63.6% (35.4-84.8); n = 7	<.001
30-day mortality, % (95% CI)	1.4% (0.7-2.9); n = 7	0% (0-2.3); n = 0	1.8% (0.9-4.0); n = 6	9.1% (1.6-37.7); n = 1	.040
120-day mortality, % (95% CI)	2.4% (1.4-4.2); n = 12	1.2% (0.3-4.3); n = 2	2.5% (1.3-4.8); n = 8	18.2% (5.1-47.7); n = 2	.021
365-day mortality, % (95% CI)	3.6% (2.3-5.6); n = 18	1.8% (0.6-5.2); n = 3	3.7% (2.1-6.5); n = 12	27.3% (9.7-56.6); n = 3	.005

Abbreviations: AGI, Acute Gastrointestinal Injury; CI, confidence interval; GI, gastrointestinal; ICU, intensive care unit; IQR, interquartile range.

^a^ Need of mechanical respiratory support, continuous or intermittent, during the first to third postoperative days.

^b^ Need of continuous or intermittent intravenous inotropic and/or vasoactive drugs during the first to third postoperative days.

^c^ With debut during the first 3 postoperative days and remaining on discharge from hospital.

^d^ Modified acute kidney injury score based on creatinine values and dialysis requirement, during the first 3 postoperative days.

^e^ Data presented for n = 324 due to ICU mortality.

^f^ Data presented for n = 10 due to ICU mortality.

^g^ Data presented for n = 320 due to in-hospital mortality.

^h^ Data presented for n = 9 due to in-hospital mortality.

### Acute Gastrointestinal Injury Grade and Outcome

Two (1.2%) patients in the AGI_0_ group developed upper GI bleeding postoperatively from ulcers verified by gastroscopy, with onset days 8 and 22, respectively. Seventeen (5.2%) patients developed GI bleeding in the AGI_1_ group with onset between days 4 and 25 postoperatively. Of these, 8 had ulcers in the ventricle and/or duodenum verified by gastroscopy or autopsy and, in 3, the source of bleeding was colorectal. In the remainder, the source of bleeding was not confirmed. Furthermore, one case of perforated duodenal ulcer was diagnosed in this group on day 4 postoperatively. Of the 11 patients in the AGI_≥2_ group, 7 (63.6%) developed at least 1 GI complication. The complications that occurred, presented as the most serious in each patient, were paralytic ileus (n = 2, 18.2%), intestinal ischemia (n = 2, 18.2%), pancreatitis (n = 1, 9.1%), upper GI bleeding (n = 1, 9.1%), and retrogastric hematoma (n = 1, 9.1%). The 2 patients who developed intestinal ischemia had thrombosis in the superior mesenteric artery and required intestinal resection. The 4 patients with paralytic ileus, GI bleeding, or retrogastric hematoma were all diagnosed on the third postoperative day. The 3 patients with intestinal ischemia or pancreatitis were diagnosed on days 13 to 18. However, these 3 patients had significant symptoms from the GI tract earlier on. A higher proportion of AGI_≥2_ patients had GI complications within 30 days after surgery (*P* < .001; Table 2). Overall, 4 (0.8%) patients died due to GI complications including duodenal bleeding and complications after perforated ulcer, mesenteric ischemia, and pancreatitis; the 2 latter having an AGI grade ≥2. The AGI_≥2_ group had a longer duration of postoperative mechanical respiratory support as well as longer ICU and hospital length of stay (*P* = .006, *P* < .005, and *P* < .005, respectively; Table 2).

**Figure 3.**
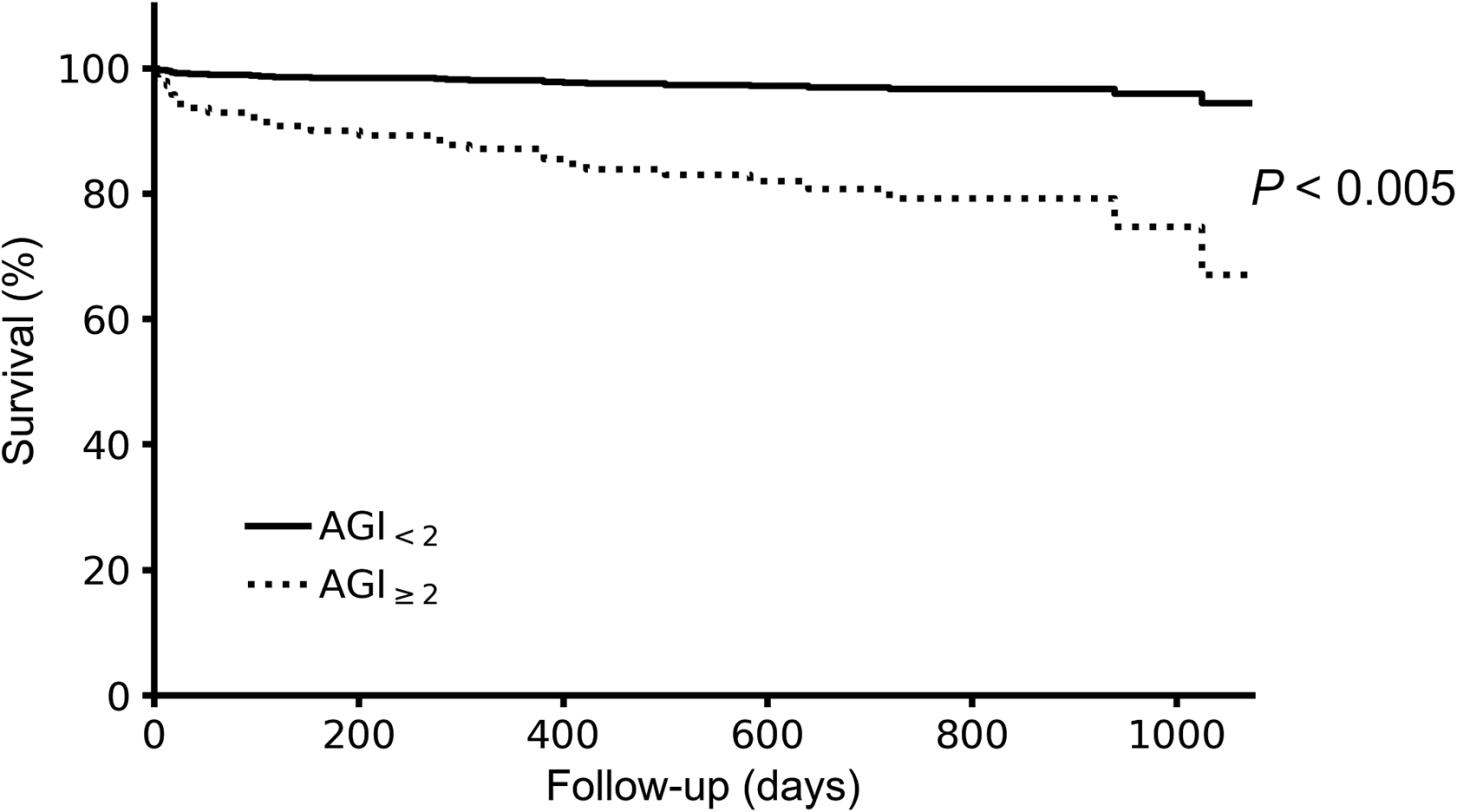
Accumulative estimated survival in relation to maximum Acute Gastrointestinal Injury grade assessed during the first 3 postoperative days in cardiac surgery patients (n = 501; *P* value derived from Cox proportional hazards regression model).

Follow-up time for investigation of mortality ranged between 365 and 1074 days. There was a higher mortality in the AGI_≥2_ group (*P* = .040 at 30 days, *P* = .021 at 120 days, and *P* = .005 at 365 days; Table 2). A multivariate Cox proportional hazards regression model was employed, including the preoperative variables in Table 1. The proportionality of baseline hazards assumption was assessed and found to be nonproportional for the contrast AGI_0_ versus AGI_1_ which was consequently removed from the model. Since univariate comparisons of survival revealed no differences when comparing AGI_0_ versus AGI_1_, AGI was reduced to a binary variable in the model, displaying the contrast AGI_≥2_ versus AGI_<2_. Of all covariates included, AGI grade ≥2 and higher EuroSCORE were associated with decreased survival (AGI_≥2_: *P* = .002; HR: 6.98, 95% CI: 1.98-24.6, EuroSCORE: *P* = .003; HR: 1.28, 95%CI: 1.09-1.51). No other of the included covariates significantly impacted survival rates (Figure 3 and Table 3).

**Table 3.** Association of AGI Grade and Preoperative Characteristics With Mortality in a Cox Proportional Hazards Model.

Variables	Hazard ratio, (95% CI)	*P*
AGI grade^a^	6.98 (1.98-24.6)	<.005
Age (years)	1.04 (0.98-1.10)	.14
Sex^b^	1.26 (0.53-3.0)	.61
Diabetes^c^	1.42 (0.58-3.49)	.44
Heart failure^c^	0.97 (0.35-2.69)	.96
Hypertension^c^	1.08 (0.45-2.60)	.87
Extracardiac arteriopathy^c,d^	1.23 (0.49-3.09)	.67
Smoking		
Quit ≥1 month ago^e^	1.21 (0.54-2.76)	.64
Active smoker^e^	1.37 (0.25-7.51)	.72
NYHA II^f^	1.14 (0.14-9.49)	.91
NYHA III^f^	2.05 (0.26-16.1)	.50
NYHA IV^f^	1.23 (0.07-22.6)	.89
EuroSCORE	1.28 (1.09-1.51)	<.005

Abbreviations: AGI, Acute Gastrointestinal Injury; CI, confidence interval; EuroSCORE, European System for Cardiac Operative Risk Evaluation; NYHA, New York Heart Association Functional Classification.

^a^ 0 = AGI_<2_, 1 = AGI_≥2_.

^b^ 0 = male, 1 = female.

^c^ Binary variable where 0 = no occurrence.

^d^ 1 = claudication, carotid artery stenosis or previous or planned surgery on abdominal aorta, and carotid arteries or distal arteries or cerebral vascular disease.

^e^ Contrasted to no smoking.

^f^ Contrasted to NYHA I.

## Discussion

The main finding of this study was that cardiac surgery patients who developed GI dysfunction (ie, a maximum AGI grade of ≥2) during the first 3 postoperative days had a statistically significant increase in GI complications and death during the follow-up period.

The AGI grading system has been evaluated in patients in a general ICU setting and mortality rate increased with higher AGI grade.^2,3^ A distinction between GI dysfunction and failure is important, since one study found a significantly higher mortality in AGI 3-4 versus AGI 1-2.^15^ It has been suggested that patients with critical illness and GI dysfunction in the absence of a primary GI cause, that is, a secondary AGI, have a higher mortality compared with those who have a primary AGI.^16^ In the current study, patients were scored during the 3 first postoperative days. In a large retrospective study, GI complications were diagnosed 9.2 ± 5.9 days after cardiac surgery with ECC, of which 58.5% primarily had an uneventful postoperative course.^17^ An extended observation period may, therefore, be beneficial, especially in patients on high dependency units. The importance of monitoring the GI tract in prolonged postoperative intensive care is further supported by a study on 320 patients requiring intensive care due to multiple organ failure after cardiac surgery, in which 10% had mesenteric ischemia.^18^ In the present study, GI dysfunction was associated with both longer ICU and hospital length of stay. A causative relationship cannot be established in the present study and GI dysfunction may coexist with other organ dysfunctions. Most probably, the total burden of intraoperative variables and postoperative morbidity associates with postoperative outcome measures. However, the present data suggest that GI dysfunction, and a structured assessment thereof, may have an important role in the postoperative course of cardiac surgery patients, a finding that should be investigated in a larger patient population. In a recent study, patients with a predicted ICU length of stay over 24 hours after cardiac surgery, an AGI grade >1.5 during the first 24 hours predicted the occurrence of multiple organ failure, infectious complications, and an ICU stay longer than 4 days.^19^ The study only comprised 40 high-risk patients, that is, a selected population, but is, to the best of our knowledge, the only published study on AGI scoring following cardiac surgery.^19^

Associations between patient characteristics and the AGI grade were found. There was a significant sex difference between the 3 AGI groups. It is well recognized that PONV is more common in women.^9^ Female sex is not an established risk factor, but mortality rates after GI complications seem to be higher in women.^4^ Extracardiac arteriopathy is a known risk factor for GI complications and it was more common in the AGI_≥2_ group.^7^ Even if EuroSCORE was associated to mortality, there was no significant difference between the 3 AGI groups in EuroSCORE. This is in line with previous findings when EuroSCORE has shown limited ability to predict GI complications.^17,20^

The aim was not to elucidate pathophysiological mechanisms of postoperative GI dysfunction. However, the proportion of thoracic aortic surgery in the AGI_≥2_ group was significantly higher than in the other groups. This may be due to the hypothermic circulatory arrest, known to increase the risk of complications.^21,22^ After elective aortic hemiarch reconstruction, a combined GI bleeding and ischemia rate of 3.3% to 3.9% was shown.^23^ Likewise, the longer duration of surgery and longer time on ECC in the AGI_≥2_ group can be attributed to the difference in types of surgery. The ECC time is an established risk factor for GI complications.^24,25^

The present study provides evidence for the hypothesis that GI dysfunction precedes GI complications since more than 60% of the patients in the AGI_≥2_ group later developed GI complications. The overall incidence of acute mesenteric ischemia was 0.4%, which is in line with previous studies.^4,6^ Furthermore, an overall high occurrence (4.4%) of GI bleeding was noted, attributable to the definition adopted and the long follow-up time.

The limitations of this study are several. The study encompasses an elective, low- to moderate-risk population, leading to few events. It is important to consider the low number of events and that only preoperative variables were included when interpreting the multivariate regression model. The material is too small to answer if AGI grade has an independent predictive value. Various definitions of GI complications have been used making comparisons between studies precarious. The AGI grade entails some signs and symptoms that also can be classified as a complication, that is, GI bleeding and to some extent bowel paralysis, which affects interpretation of the results. The chosen marker of circulatory status, number of inotropic, and/or vasoactive drugs used is a crude estimation, as a precise definition of different circulatory dysfunctions in most situations following cardiac surgery is missing. The need of drugs is, however, easily obtained and accurately recorded. Also, ideally a more precise measurement, such as a scale for delirium, could have been used coincidently with the one employed for neurologic assessment. Furthermore, a validated daily compound organ dysfunction score for use after cardiac surgery would have been informative, but to our knowledge, such a score is lacking.

## Conclusions

Early postoperative GI dysfunction, measured as maximum AGI grade ≥2 during the first 3 days following cardiac surgery, was associated with higher incidence of postoperative GI complications within 30 days and higher mortality. In addition, postoperative duration of mechanical respiratory support, and length of ICU and hospital stay were longer in patients with early postoperative GI dysfunction. Increased attention to early GI dysfunction in cardiac surgery patients is warranted and the AGI grade could be a helpful adjunct to a structured approach.
